# Antibacterial Activity of *Syzygium aromaticum* (Clove) Bud Oil and Its Interaction with Imipenem in Controlling Wound Infections in Rats Caused by Methicillin-Resistant *Staphylococcus aureus*

**DOI:** 10.3390/molecules27238551

**Published:** 2022-12-05

**Authors:** Abdulaziz Khaleef Alanazi, Mohammed Hussein Alqasmi, Mohammed Alrouji, Fahd A. Kuriri, Yasir Almuhanna, Babu Joseph, Mohammed Asad

**Affiliations:** 1 Department of Clinical Laboratory Sciences, College of Applied Medical Sciences, Shaqra University, Shaqra 11961, Saudi Arabia; 2 Medical Laboratory, Hafar Albatin Central Hospital, Hafar Albatin 39513, Saudi Arabia

**Keywords:** excision wound, epithelization, essential oils, wound healing

## Abstract

Methicillin-resistant *Staphylococcus aureus* (MRSA) is one of the leading causes of infection worldwide. Clove oil’s ability to inhibit the growth of MRSA was studied through in vitro and in vivo studies. The phytochemical components of clove oil were determined through gas chromatography-mass spectrometry (GC-MS) analysis. The antibacterial effects of clove oil and its interaction with imipenem were determined by studying *MIC*, MBC, and *FIC* indices in vitro. The in vivo wound-healing effect of the clove oil and infection control were determined using excision wound model rats. The GC-MS analysis of clove oil revealed the presence of 16 volatile compounds. Clove oil showed a good antibacterial effect in vitro but no interaction was observed with imipenem. Clove bud oil alone or in combination with imipenem healed wounds faster and reduced the microbial load in wounds. The findings of this study confirmed the antibacterial activity of clove oil in vitro and in vivo and demonstrated its interaction with imipenem.

## 1. Introduction

*Staphylococcus aureus* is a commensal microbe that often causes wound infections [1]. It is a multidrug-resistant organism that colonizes skin, wounds, the nose, the perineum, and the throat, leading to infections in various organs [2]. Approximately one in ten hospital admissions worldwide results in hospital-acquired infections and of these 8% are caused by MRSA [3]. 

The hunt for novel antibacterial chemicals including herbal products is rapidly increasing in order to produce better medications for combating multidrug resistance organisms [4]. Medicinal products derived from aromatic plants are widely used in traditional medicines for controlling bacterial infections [5]. Many reports indicate that antibiotic-resistant strains can be controlled by using essential oils [6]. These oils are highly volatile, hydrophobic, and lipophilic with numerous biological and pharmacological properties [7]. 

*Syzygium aromaticum*, commonly called clove, (family-Myrtaceae), is indigenous to the islands of Indonesia. It is now cultivated worldwide as a flavoring agent, for medicinal purposes, and for use in perfumes [8]. It is widely used as a food preservative due to its antimicrobial properties [9]. Apart from its antimicrobial properties, antioxidant, analgesic, anesthetic, anti-inflammatory, and insecticidal activity have been reported [10]. It is also used for preventing degenerative diseases because of its antioxidant effects [11]. Clove bud oil is a common healing agent for wounds and burns in traditional medicine [12]. The antibacterial and wound infection control efficacy of clove bud oil and its interaction with imipenem were determined in the present study. A detailed phytochemical screening was also carried out by gas chromatography-mass spectrometry (GC-MS) to detect volatile components in the oil.

## 2. Results

### 2.1. Gas Chromatography-Mass Spectrometric Analysis

**Figure 1:**
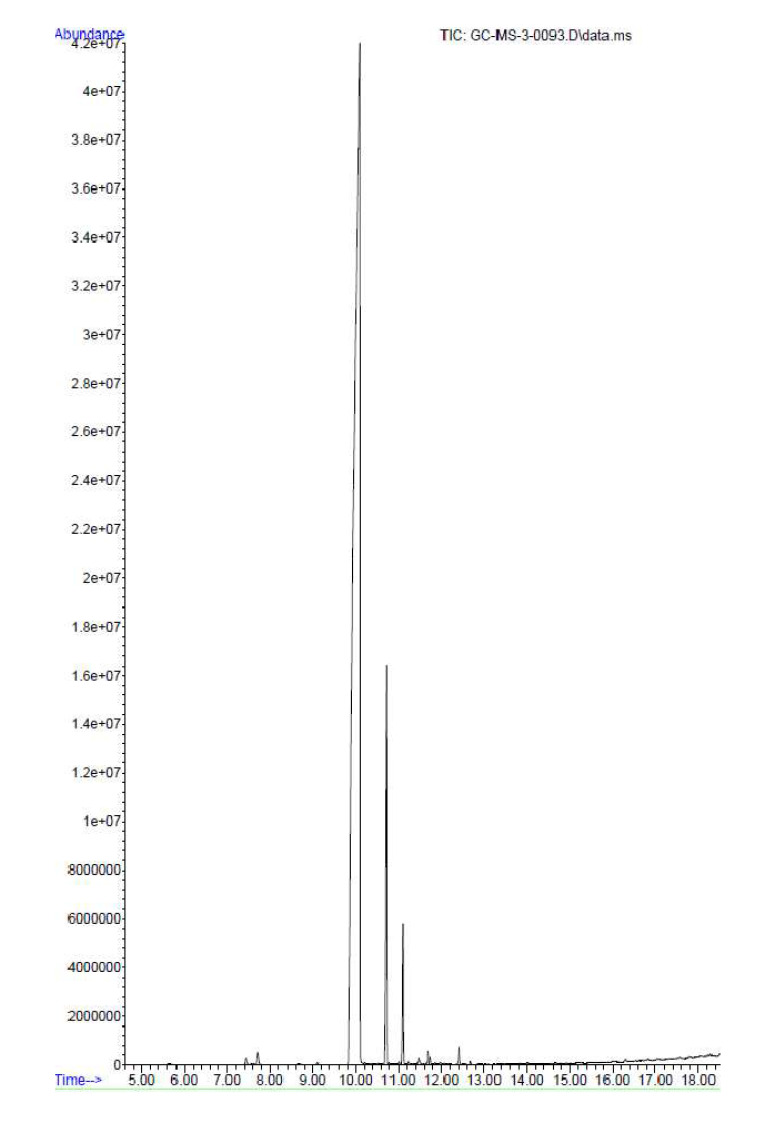
GC-MS spectra of clove oil.

The clove oil was subjected to silylation for the derivatization of constituents to facilitate GC-MS analysis. The spectrum produced via gas chromatography is shown in Figure 1. The list of compounds detected is shown in Table 1. Eugenol, caryophyllene, and 2-(octadecyloxy)-ethanol were the major compounds identified by the GC-MS analysis. The calculated retention indices and their comparison with the retention indices from the literature, with references included, are given as supplementary data (Table S1).

### 2.2. Antibacterial Activity In Vitro

Clove oil showed good antibacterial effects at 20 µL/mL and 40 µL/mL against MRSA. The zone of inhibition was not affected when it was combined with imipenem (Table 2). Concentrations of less than 20 µL/mL showed a much lower inhibition zone.

The MRSA strain showed a resistant pattern toward most of the antibiotics, as shown in Table S2. It was sensitive to linezolid, vancomycin, and minocycline. An intermediate degree of sensitivity was observed for imipenem, and this was selected as a standard antibiotic for the study (Table S1).

The *MIC* of clove oil was found to be 2.5 µL/mL and a concentration of 5 µL/mL was found to be the MBC against MRSA. There were no colonies formed on the mannitol salt agar when a loop of culture was inoculated from the tubes containing 5 µL/mL clove oil and above (Table 3).

The clove oil showed an antibacterial effect when tested separately; however, there were no antagonistic or synergetic effects when it was combined with imipenem (Table S2).

### 2.3. Wound-Healing and Skin Irritation Study

The period of epithelization of the wounds was determined on each day after the application of the ointment formulation. The inoculation of wounds with MRSA significantly reduced the epithelization period. 

**Figure 2:**
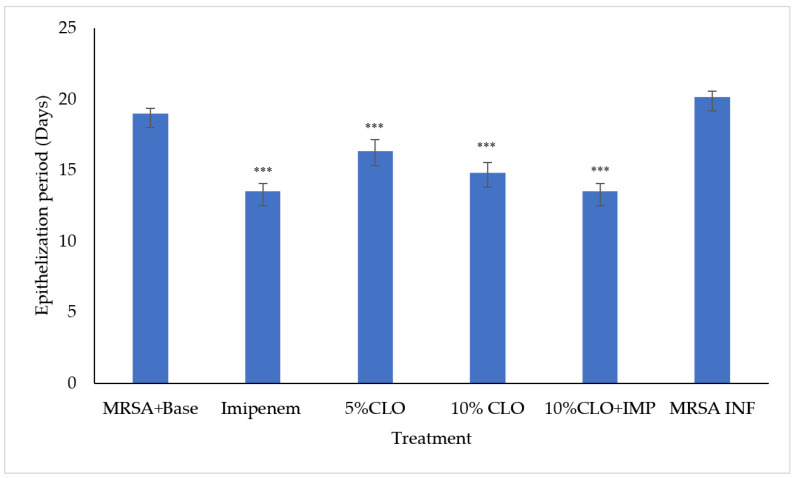
Epithelization period after different treatments. Values are mean±SEM for six animals; *** *p* < 0.001 in comparison to MRSA+base (CLO—clove oil, IMP—imipenem, and MRSA INF—MRSA infected).

The wounds were healed, and epithelization was significantly quicker after different treatments in comparison to the control (Figure 2). Of all the treatments, the lowest period of epithelization was observed in the combination group followed by imipenem. The effect of 10% clove oil was comparable to that observed with imipenem. The lower concentration of clove oil (5%) was the least effective. However, there was no significant difference between the different concentrations of clove oil and imipenem, indicating a lack of dose-dependent response or interaction between imipenem and clove oil.

**Figure 3:**
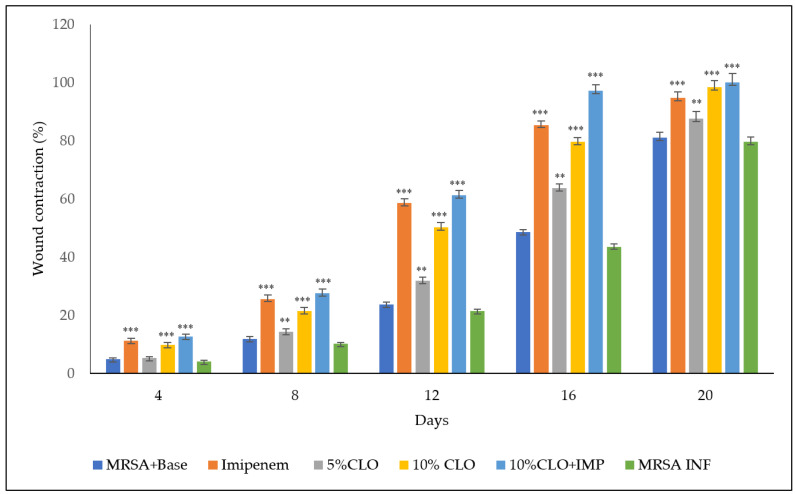
Wound contraction (%) after different treatments. Values are mean ±SEM for six animals; ** *p* < 0.01 and *** *p* < 0.001 in comparison to MRSA+base (CLO—clove oil. Imp—imipenem, and MRSA INF—MRSA-infected).

**Figure 4:**
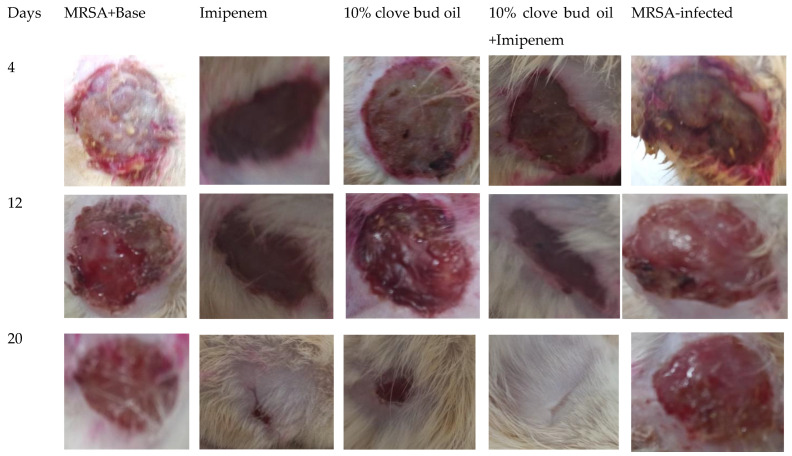
Images of wounds infected and treated with different concentrations of clove oil and imipenem.

The degree of wound contraction was measured at 4-day intervals for up to 20 days. The MRSA strain aggravated the wounds, leading to fluid exudation from the wounded tissues in all the animals with few deaths (two out of eight animals) in the control group. The measurements obtained from the six animals throughout the treatment period were ensured by adding more animals in the groups where mortality was observed. The clove oil (5 % *w*/*w*) did not show any significant change in wound healing on day 4, while it was less effective than clove oil (10% *w*/*w*) and imipenem in contracting the wounds on the other days of measurement (Figure 3). However, this difference was not significant. The clove oil (10% *w*/*w*) and imipenem improved the healing of wounds from the 4 day onwards. A similar result was observed in the combination group of clove oil 10% (*w*/*w*) and imipenem (Figure 4).

**Figure 5:**
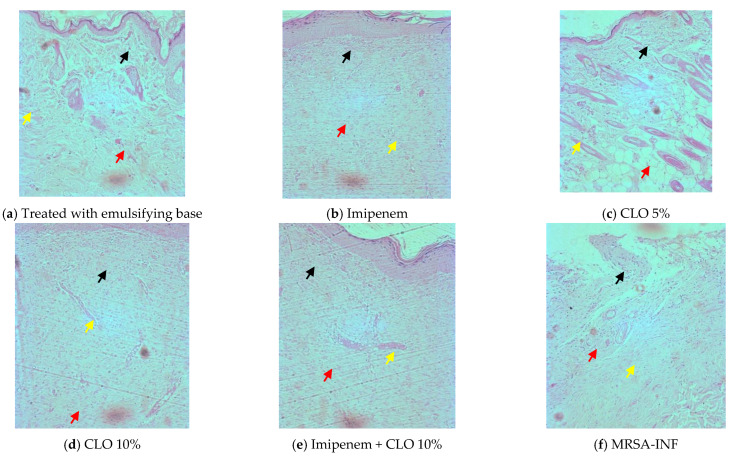
Photomicrographs indicate wound healing after different treatments. Epidermis (black arrow); capillaries (yellow arrow); inflammatory cells (red arrow). (CLO—clove oil, MRSA INF—MRSA-infected).

In the histological examination, a worn-out epidermis was seen in the skin sections of the control animals. Of all the treatments, the imipenem and clove oil combination showed better healing followed by clove oil (10% *w*/*w*) and clove oil (5% *w*/*w*). The epidermal layer was damaged in the control animals infected and not subjected to treatments, with a large bulk of inflammatory cells and fewer capillaries. Imipenem and clove oil (10% *w*/*w*) and their combination improved epidermal regeneration and angiogenesis (Figure 5).

The log CFU was significantly lower in all the treatment groups when compared to the control group with respect to all treatments. No significant difference was seen between the imipenem-treated group and the group that received a combination of imipenem and clove oil (10%). This indicates a lack of interaction between clove oil and imipenem (Table 4).

No noticeable redness (erythema) or inflammatory reaction was seen until 3 days after the application of the clove oil formulation in the skin irritation test.

## 3. Discussion

The present study examined the outcome of clove oil treatment on different features of wound healing. The determination of the antibacterial action showed its effect against MRSA, which is known to infect wounds. The histological study on the wounded tissues was carried out to support clove oil’s wound-healing activity and infection control ability. The GC-MS analysis revealed the various chemical constituents present in the oil. The major constituents of essential oils evaporate above 60 °C [23]. Gas chromatography is used to separate volatile compounds; hence, GC-MS analysis was carried out to identify the different volatile compounds present in the clove essential oil. The silylation procedure was carried out to mask the polar groups that may be present in the components of essential oil. Silylation is the most common procedure used to reduce the polarity of analytes and improve their stability for gas chromatography. Apart from this, other procedures such as alkylation are used. However, there are no reports suggesting that one method is superior to the other for the derivatization of essential oils. The mass spectra obtained were used to identify compounds via the NIST library. The RI values of the marked compounds in Table 1 are for silyl derivatives, as these were derivatized by silylation. The RI values from the literature were taken from the NIST libraries. The RI from the literature was selected by distinguishing the closed match with our calculated RI values.

Clove oil has been reported to possess several biological activities, including antibacterial, anti-inflammatory, analgesic, antioxidant, fungicidal, and antitumor activities [24]. Clove oil contains several active constituents, including pinene (α and β), neral, geranial, γ-terpinene, cis-ocimene, allo-ocimene, 1,8-cineole, linalool, borneol, myrcene, and pinene-2-ol [24]. In the present study, 16 components were identified by GC-MS analysis. Eugenol, caryophyllene, and 2-(octadecyloxy)-ethanol were the major constituents. All these constituents have been previously reported [25].

There are several bases available for the formulation of ointments; the emulsifying base prepared following the British pharmacopeial method was used in the present study [26]. To demonstrate that the base used for the preparation of the ointment is inert, a separate group (MRSA-infected) was established, and this group was compared with the MRSA control, which was administered with the base only. No significant difference in wound healing was observed and this demonstrated the inertness of the base. The diffusion of active antimicrobial compounds from the clove oil ointment formulation was confirmed by studying the passage through the Muller Hinton agar medium (data not shown). The microorganism used for wound infection was chosen after a literature review in order to produce the most challenging infection [27]. Clove oil has been reported to possess antibacterial activity against MRSA [28]. Imipenem served as positive control and the selection was made based on an antibiotic sensitivity test where an intermediate degree of sensitivity was observed for this antibiotic against MRSA. The pathogen showed resistance to amikacin [29], amoxicillin/k clav [30], ampicillin [31], ceftaroline [32], ciprofloxacin [33], clindamycin [34], erythromycin [35], gentamicin [36], levofloxacin [37], oxacillin [38], tetracycline [39], and trimethoprim/sulfa [40].

Earlier studies on essential oils reported antibacterial effects against Gram-negative bacterial strains causing urinary tract infections [41]. The anti-MRSA potential of clove oil has also been reported and has highlighted the need to explore bioactive constituents [42]. The phytochemicals present in clove bud oil are responsible for antimicrobial activity. Eugenol is one of the phytoconstituents that might have significantly contributed to the antimicrobial effects [43]. Eugenol is a primary constituent of clove but it is also found in cinnamon, pepper, and *Ocimum sanctum* [44]. Another constituent that is responsible for both antibacterial and wound-healing properties is caryophyllene [45,46]. Apart from clove, several other plants, such as basil (*Ocimum* spp.), cannabis, lavender, and black pepper, contain caryophyllene [47]. Other constituents such as levomenthol are used in mouthwashes for antimicrobial effects [48]. However, levomenthol is the main constituent of *Mentha piperita* [49], and is found only in smaller amounts in clove. Humulene is another constituent and is a cannabis terpene similar to caryophyllene with antibacterial effects [50]. However, the minor components might also contribute considerably by merging with other major components for controlling the growth of MRSA. The hydrophobic nature of the oil may also help in interacting with the outer cytoplasmic membrane of MRSA and affects the integrity and the functioning of the cell membrane [51].

Each of the parameters, such as wound contraction and the histological studies, indicates a stage of healing. Clove oil effectively increased the healing of excision wounds without causing any noticeable irritation to the skin. Each of the parameters used in the present study discloses factors that contributed to the wound-healing action. The wound contraction indicates improvement in wound closure while the complete healing of the wound is shown by the fading of the scar, which was taken to be the period of epithelization [52]. The clove oil formulation increased the healing of wounds alone as well as in combination with imipenem in the infected wounds, though the effect produced by the combination was not significantly different from either of the treatments alone. Histological studies were carried out to confirm the results of the macroscopic observations. Cellular organelles can be easily visualized after staining with H and E [53]. The epidermis is the outermost layer of the skin, and its thickness shows the regeneration of the epithelial lining. The number of capillaries indicates the increased formation of blood vessels that are required in the wound-healing process. Inflammation is an early reaction to an injury, and it is mediated through various inflammatory cells that include macrophages, neutrophils, and plasma cells. However, the presence of inflammatory cells in the wounded tissue several days after wounding shows incomplete wound healing [54]. An increase in epidermal height and capillary number and a decrease in inflammatory cells after the clove oil treatment showed increased healing of wounds.The fast and rapid wound healing activity of clove oil may be due to the antioxidant and anti-inflammatory activities of its phytochemical contents in addition to its antibacterial effect, especially with respect to phenolic compounds [55,56]. However, there may be additional mechanisms that have to be explored. A study of the individual components of the clove and their effects in combination may provide more insight into the mechanism(s) underlying their wound-healing action, contribution, and/or interaction towards this effect. 

## 4. Materials and Methods

### 4.1. GC-MS Analysis of Clove Oil

A GC-MS 7890A GC system with 5975C VL MSD from Agilent Technologies (California, USA) was used. Clove oil (100 µL) was mixed with 250 µL of water and 750 µL of ethyl acetate. Following this, the upper layer was separated and concentrated. To this, a 50 µL mixture of N, O-Bis (trimethylsilyl) trifluoroacetamide (49.5 µL) and trimethylchlorosilane (0.5 µL) was added followed by the addition of pyridine (10 µL). This was heated for 30 min at 60 ºC and the contents were dried using liquid nitrogen before finally dissolving the dried sample (20 mg) in methanol (5 ml) for analysis. After filtration through a membrane filter (0.22 µm), 3 µL was injected through a capillary column (30 m, 0.25 mm, and 0.25-microns) with an injector temperature of 270 °C and pressure at 80 kPa. The carrier gas was hydrogen and the total time for analysis was 25 min. 

For determination of calculated kovat’s retention index, a homologous series of n-alkanes were injected into the same GC-MS operating system. The retention indices of isolated constituents were determined using the following formula [57]:

I = 100[n + (N − n) × (logt_r_ (unknown) − logt_r_ (n))/logt_r_(N) − logt_r_(n)) (1)

where

I = Kovat’s index;

n= number of carbon atoms in smaller n-alkane;

N= number of carbon atoms in larger n-alkane;

t_r_ = retention time.

The referenced Kovat’s retention indices were obtained from the NIST library. The calculated Kovat’s retention indices were matched with those from the reported literature.

### 4.2. Antibiotic Sensitivity Test

MRSA isolates available in the laboratory (ATCC43300) were tested for drug resistance and a methicillin-resistant organism was selected for the study. The antibiotic sensitivity test for the determination of multidrug resistance was carried out using the Microscan system. The trays were read by using an autoSCAN-4 reader at 620, 560, 505,470, 440, and 590 nm, and the results were recorded.

### 4.3. Antibacterial Assay (Well Diffusion Assay)

The antibacterial evaluation of clove oil was carried out by the well diffusion method. The wells were loaded with oil dissolved in dimethylsulfoxide (DMSO) [58]. DMSO (10 % *v*/*v*) served as a control. The zone of inhibition was measured after incubation at 37 °C for 24 h.

### 4.4. Minimum Inhibitory Concentration (MIC) and Minimum Bactericidal Concentration (MBC)

Muller Hinton broth was inoculated with MRSA containing 1.5 × 10^8^ CFU/mL (0.5 McFarland standard turbidity). The *MIC* and MBC were determined from the final concentration of clove oil (1.25, 2.5, 5, 10, 20, and 40 µL/mL) in broth. The minimum concentration of clove oil that inhibited the visible growth was taken as *MIC*. The minimum concentration at which no bacterial growth was seen after culturing was taken as MBC. 

### 4.5. Interaction of Clove Oil with Imipenem against MRSA

Antagonistic or synergetic assay of the oil with imipenem was determined by broth dilution method using checkerboard assay [59]. Different concentrations of antibiotic and oil were used. The fractional inhibitory concentration (*FIC*) index was determined using the following formulae:

*FIC index* = *FIC _oil_* + *FIC _antibiotic_*

*FIC _oil_* = *MIC _oil *+* antibiotic_*/*MIC _oil_*

*FIC _antibiotic_* = *MIC _oil *+* antibiotic_*/*MIC _antibiotic_*

*FIC index* of ≤ 0.5 indicates the synergetic effect of the combination; values between 0.5 and ≤ 2 suggest that the combination is indifferent; and an *FIC index* > 2 shows that the combination is antagonistic [60].

### 4.6. Wound-Healing Activity in Rats (Excision Wound Model)

Albino Wistar rats of either sex weighing between 220 to 232 g and maintained under controlled conditions were utilized for this study. All procedures performed on the animals followed ARRIVE guidelines. The Ethical Research Committee of Shaqra University approved the study (Approval number—53/18910). Persons handling the animals exercised the utmost care to prevent the transmission of MRSA infection. All parameters were measured by individuals unaware of the treatment. Emulsifying bases prepared by mixing soft paraffin (50%), liquid paraffin (20%), and emulsifying wax (30%) were used to formulate clove oil ointment [61]. Two different concentrations (5% *w*/*w* and 10% *w*/*w*) of clove oil were employed. Physicochemical properties, stability, and diffusion of the prepared ointment were tested [62]. The emulsifying base was applied to control animals.

Animals were divided into six groups (n = 6):

Group I—Control (emulsifying base);

Group II—Imipenem;

Group III—Clove oil (5% *w*/*w* in emulsifying base);

Group IV—Clove oil (10% *w*/*w* in emulsifying base);

Group V—Clove oil (10% *w*/*w* in emulsifying base) + imipenem;

Group VI—Infected control (MRSA).

To inflict the excision wounds, the dorsal thoracic region of the animals was shaved after anesthetizing them with a combination of ketamine (91 mg) and xylazine (9.1 mg) at a dose of 1 mL/kg administered intraperitoneally [63]. A circular area (500 mm^2^) was marked and this was excised to full thickness [64]. The broth culture MRSA (1.5 × 10^8^ CFU/mL) was inoculated into the freshly excised wound [65]. Animals were given different treatments, as mentioned above, once per day starting one day after inoculation of MRSA. To prevent the biting of wounds, animals were individually caged. The formulation was prepared and applied to the wounds. The wound area was determined every 4 days until the formed scab fell off. Wound contraction (%) was determined in each group. The day the scabs fell off was considered the day of epithelization. The healed area of the wound was subjected to histological examination and one for determination of CFU/g tissue. The tissue samples (1 g) were collected on the final day of the experiment and homogenized for 5 min using phosphate buffer saline (1 mL) via an aseptic technique. Homogenates were serially diluted (up to 10^9^) and plated on nutrient agar. Plates were incubated at 37 ºC and the colonies were counted accordingly. 

### 4.7. Skin Irritation Test 

Clove oil ointment was applied to the shaved skin on the dorsal side of the rats followed by the area’s covering with adhesive tape. Skin reactions that manifested as redness or inflammation (if any) were observed every 12 h for three days [66]. 

### 4.8. Statistical Analysis

The mean ± SEM values were used to analyze statistical significance by one-way ANOVA with Tukey’s post hoc test (SPSS version 20 for Windows).

## 5. Conclusions

The clove oil showed good antibacterial activity against MRSA in vitro with an *MIC* of 1.25 µL /ml and an MBC of 2.5 µL/mL, though no interaction was observed with imipenem. The results regarding the in vitro antibacterial effects were further confirmed by a significant reduction in microbial load in the excision-wound-healing model rats. The GC-MS analysis of the clove oil showed the presence of 16 volatile components that have been previously reported to possess antibacterial effects.

## Figures and Tables

**Table 1:** Major components identified by GCMS of *S. aromaticum* oil.

Peak	Name	Retention Time	Area (%)	RI _cal_	RI _lit_	Reference
1.	Cyclohexanone, 5-methyl-2-(1-methylethyl)-, trans-	7.431	0.13	1114	1124	[13]
2.	* Levomenthol	7.709	0.28	1123	1143.95	[14]
3.	* Phenol, 4-(2-propenyl)-	8.664	0.05	1151		--
4.	* Eugenol	10.097	72.54	1388	1378	[15]
5.	Caryophyllene	10.731	5.16	1405	1392.14	[14]
6.	Humulene (α- Caryophyllene)	11.097	1.55	1415	1428	[16]
7.	Bicyclosesquiphellandrene	11.219	0.06	1519	1521	[17]
8.	α-Farnesene	11.475	0.13	1525	1522	[18]
9.	Naphthalene, 1,2,3,5,6,8a-hexahydro-4,7-dimethyl-1-(1-methylethyl)-, (1S-cis)-	11.686	0.25	1531	1528	[19]
10.	Caryophyllene oxide	12.408	0.22	1538	1537	[20]
11.	* 1-Decanol, 2-hexyl	15.163	0.09	1492	1504	[21]
12.	Heptadecane	15.741	0.15	1719		--
13.	Hexatriacontyl pentafluoropropionate	17.308	0.50	1962		--
14.	Heptadecane, 2,6,10,15-tetramethyl-	17.563	0.59	2168		--
15.	1-Docosene	17.774	0.051	2179	2190	[22]
16.	* 2-(octadecyloxy)ethanol	18.285	1.00	1284		--

* Indicates compounds were derivatized by silylation.

**Table 2:** Antibacterial activity of clove oil and its combination with imipenem against MRSA.

Antibacterial Agent	Zone of Inhibition (mm)
Clove oil (20 µL/mL)	10
Clove oil (40 µL/mL)	13
Imipenem (4 µL/mL)	22
Imipenem (4 µL/mL) + Clove oil (40 µL/mL)	22

Values are replicates from three measurements.

**Table 3:** *MIC* and MBC of clove oil against MRSA.

Clove Oil (µL/mL)	Visible Growth (*MIC*)	Growth on Mannitol Salt Agar (MBC)
20	–	–
10	–	–
5	–	–
2.5	–	+
1.25	++	+
0.62	++	+
0.31	++	++

+ indicates visible growth, ++ indicates more growth and – shows no observed growth.

**Table 4:** The bacterial load (CFU/g) in the skin tissue after treatment.

Treatment	Bacterial Load (Log_10_ CFU/g of Tissue)
MRSA+base	10.34 ± 0.49
MRSA-infected + Imipenem	4.11 ± 0.48 ***
MRSA-infected + 5% CLO	5.89 ± 0.47 ***
MRSA-infected + 10% CLO	3.95 ± 0.33 ***
MRSA-infected + imipenem + 10% clove oil	3.1 ± 0.32 ***
MRSA-infected wounds	11.07 ± 0.41 ^ns^

Values are mean ± SEM for six animals *** *p* < 0.001, ^ns^ Non-significant change in comparison to MRSA+base (CLO—clove oil).

## Data Availability

Not applicable.

## Supplementary Materials

The following supporting information can be downloaded at: https://www.mdpi.com/article/10.3390/molecules27238551/s1, Table S1: Antibiotic resistance profile of MRSA; Table S2. The fractional inhibitory concentration of clove oil against MRSA.
