# Supplementary material for: Antibacterial Activity of Syzygium aromaticum (Clove) Bud Oil and Its Interaction with Imipenem in Controlling Wound Infections in Rats Caused by Methicillin-Resistant Staphylococcus aureus
Source: Molecules. 2022 Dec 5;27(23):8551. doi: 10.3390/molecules27238551 (PMC9736006; doi:10.3390/molecules27238551)
Supplement: Supplementary file 1 [file molecules-27-08551-s001.zip › molecules-1967735-supplementary.pdf]

Table S1. Antibiotic resistance profile of MRSA

Antibiotic	Concentration (mcg/ml)	Result
Amikacin	32	Resistant
Amoxicillin/K Clav	4	Resistant
Ampicillin	8	Resistant
Ceftaroline	1	Resistant
Ciprofloxacin	2	Resistant
Clindamycin	2	Resistant
Erythromycin	4	Resistant
Gentamycin	8	Resistant
Imipenem	2	Intermediate
Levofloxacin	4	Resistant
Linezolid	1	Sensitive
Minocycline	1	Sensitive
Oxacillin	2	Resistant
Tetracycline	8	Resistant
Trimethoprim/Sulfa	4/76	Resistant
Vancomycin	1	Sensitive

Table S2. The fractional inhibitory concentration of clove oil against MRSA

Bacterial pathogens	Fractional inhibitory concentration				Remarks
	MIC Oil (µl/ml)	MIC of Imipenem (mg/ml)	MIC of Imipenem +oil	FIC Index	
MRSA	2.5	0.004	0.004	1.016	Indifference
